# Supplementary material for: Deciphering oxidative stress contributions in vestibular schwannoma: A bioinformatics approach to novel therapeutic pathways
Source: PLoS One. 2025 Nov 13;20(11):e0336451. doi: 10.1371/journal.pone.0336451 (PMC12614553; doi:10.1371/journal.pone.0336451)
Supplement: Attachment — Submitted filename: Response to Reviewers.docx [file pone.0336451.s006.docx]

**Manuscript ID: PONE-D-25-46280**

**Title:** Deciphering oxidative stress contributions in vestibular schwannoma: a bioinformatics approach to novel therapeutic pathways

Response to Reviewer #2

Comment 1 (Figure 4A–F resolution):

We thank the reviewer for the careful evaluation. Figure 4A–F was already re-exported at 600 dpi TIFF with uniform font sizes (≥8 pt) and line weights (≥0.5 pt) during the previous revision, and it successfully passed the PACE preflight check under the PLOS ONE preset. We have reconfirmed that the submitted Figure 4 file is in TIFF format, RGB mode, and 600 dpi resolution (3984 × 4226 px), which fully meets the journal’s requirements.

Comment 2 (Figure 7B resolution):

We respectfully note that Figure 7B was also re-exported at 600 dpi with adjusted typography in the previous revision, and it passed the PACE preflight check at that time. We have reconfirmed that the submitted Figure 7 file is in TIFF format, RGB mode, and 600 dpi resolution (4007 × 1851 px), which fully meets the journal’s standards. No further changes are required.

Comment 3 (Data availability):

We apologize for any confusion. In addition to the initial deposition at Zenodo (concept DOI: 10.5281/zenodo.17163233), we have now created an updated version including all per-figure underlying data and the full R scripts (version DOI: 10.5281/zenodo.17312999). The Data Availability Statement has been revised accordingly to ensure full transparency and reproducibility.
